# A descriptive study of a Smoke-free Teens Programme to promote smoke-free culture in schools and the community in Hong Kong

**DOI:** 10.1186/s12889-018-6318-4

**Published:** 2019-01-07

**Authors:** Oi Kwan Chung, William Ho Cheung Li, Ka Yan Ho, Antonio Cho Shing Kwong, Vienna Wai Yin Lai, Man Ping Wang, Katherine Ka Wai Lam, Tai Hing Lam, Sophia Siu Chee Chan

**Affiliations:** 1 School of Nursing, The University of Hong Kong, 4/F, William M.W. Mong Block, 21 Sassoon Road, Pokfulam, Hong Kong, People’s Republic of China; 2 The Hong Kong Council on Smoking and Health, Hong Kong, People’s Republic of China; 3 School of Public Health, The University of Hong Kong, Hong Kong, People’s Republic of China

**Keywords:** Ambassadors, Attitudes, Knowledge, Practice, Tobacco control, Youth smoking

## Abstract

**Background:**

Youth smoking continues to be a significant global public health concern. To ensure healthier lives for youths, healthcare professionals need to increase awareness among the youth of the health risks and addictive nature of smoking, strengthen their ability to resist negative peer influence and curiosity, and help those who smoked to quit. The Smoke-free Teens Programme was launched in 2012 to equip youngsters with up-to-date information about smoking and global trends in tobacco control and to encourage them to play a pioneering role in tobacco control. This paper describes the process and outcomes of this programme for youths in Hong Kong.

**Methods:**

The Smoke-free Teens Programme contained three major components: (i) a 2-day-1-night training camp; (ii) creative activities to promote smoke-free messages in schools and the community; and (iii) an award presentation ceremony to recognize the efforts of outstanding Smoke-free Teens in establishing a smoke-free culture. All secondary school students or teenagers aged 14 to 18 years from secondary schools, youth centres and uniform groups were invited to join the programme. The outcome measures were changes in (1) knowledge about smoking hazards; (2) attitudes towards smoking, tobacco control, and smoking cessation; and (3) practices for promoting smoking cessation.

**Results:**

A total of 856 teenagers were recruited during the study period (July 2014 to March 2017). The results showed statistically significant changes in participants’ knowledge about smoking hazards, attitudes towards tobacco control, and practice for promoting smoking cessation.

**Conclusions:**

The Smoke-free Teens Programme demonstrated effectiveness in equipping youngsters with up-to-date information about smoking and global trends in tobacco control and in encouraging them to play a pioneering role in tobacco control. The trained Smoke-free Teens not only promoted the smoke-free messages among their schoolmates, friends, and families, but also gathered community support for a smoke-free Hong Kong. The programme has been instrumental in fostering a new batch of Smoke-free Teens to advocate smoke-free culture and protect public health.

**Trial registration:**

Clinicaltrials.gov ID NCT03291132 (retrospectively registered on September 19, 2017).

## Background

With an annual death toll of almost 7 million worldwide, cigarette smoking is the biggest preventable cause of premature death and disease [1]. Two-thirds of all premature deaths of smokers can be directly attributed to smoking, and smoking is especially hazardous for those who start smoking at a young age [2–6]. There is evidence that smoking cessation before the age of 40 can reduce the death rate by more than 90% [2]. Even though early cessation is critical to reducing the hazard that smoking poses to an individual’s health, young people who, driven by curiosity and peer pressure, begin to experiment with smoking are likely to continue the habit into adulthood [7]. Most adult smokers started smoking when they were young [8]. It is therefore essential that healthcare professionals should increase awareness among the young of the health risks and addictive nature of smoking, strength their ability to resist negative peer influence and curiosity, and help those who smoked to quit.

Over the past 30 years, enormous efforts have been made by the Hong Kong government to raise tobacco tax and introduce legislation, law enforcement, and smoke-free campaigns, which have led to remarkable success in tobacco control [9, 10]. The prevalence of daily cigarette smoking in Hong Kong decreased from 23.3% in 1982 to 10.5% in 2015 [11, 12]. Nevertheless, 641,300 people aged 15 years or above still smoke daily [12] and this remains an important public health concern in Hong Kong.

The Hong Kong Council on Smoking and Health (COSH) was established under its own ordinance in 1987. This is a statutory body vested with various functions, as set out in the Hong Kong Council on Smoking and Health Ordinance (http://smokefree.hk/UserFiles/resources/about_us/CAP_389_Eng.pdf), to protect and improve the health of the community by (1) informing and educating the public about smoking and health matters; (2) conducting and coordinating research into the cause, prevention, and cure of tobacco dependence; and (3) advising government, community health organizations, or any public body on matters related to smoking and health. Under this charter, COSH has been an active player and commentator on all issues related to tobacco control. To educate youngsters about smoking hazards and the latest trends in tobacco control, and to encourage them to play a pioneering role in spreading smoke-free messages, COSH launched the Smoke-free Teens Programme in 2012 (formerly named Smoke-free Youth Ambassador Leadership Training Programme). The objectives of the programme are to (1) equip a group of young leaders with up-to-date information about smoking hazards and global trends in tobacco control and to encourage them to play a pioneering role in tobacco control; (2) penatrate the smoke-free message into schools and the community via the Smoke-free Teens; (3) encourage the Smoke-free Teens to act as role models and develop a smoke-free healthy lifestyle; and (4) equip the young leaders with basic smoking cessation counseling skills. In fact, training teenagers to serve as behavior change agents has been widely suggested in the literature [13], and has been proven effective to combat alcohol use and substance abuse [14, 15]. Yet, this strategy has not been applied to establish a smoke-free culture. To address the gap in existing literature, this paper describes an evaluation of this programme’s effectiveness in promoting the smoke-free culture among youths. However, the evaluation was carried in relation to the first three objectives, but not the fourth. This is because, some participants might not encounter any smoker during the study period; it would be difficult for us to assess their smoking cessation counseling skills.

## Methods

The study evaluated the Smoke-free Teens Programme from July 2014 to March 2017. The study design and the procedure to obtain informed consent were approved by the Institutional Review Board of the University of Hong Kong and Hospital Authority Hong Kong West Cluster (reference UW14–412). Written consent was obtained from the participants’ parents after fully informing them of the study’s purpose and details. They were told that the participation of their child was totally voluntary and without any prejudice.

### Design and participants

**Fig. 1:**
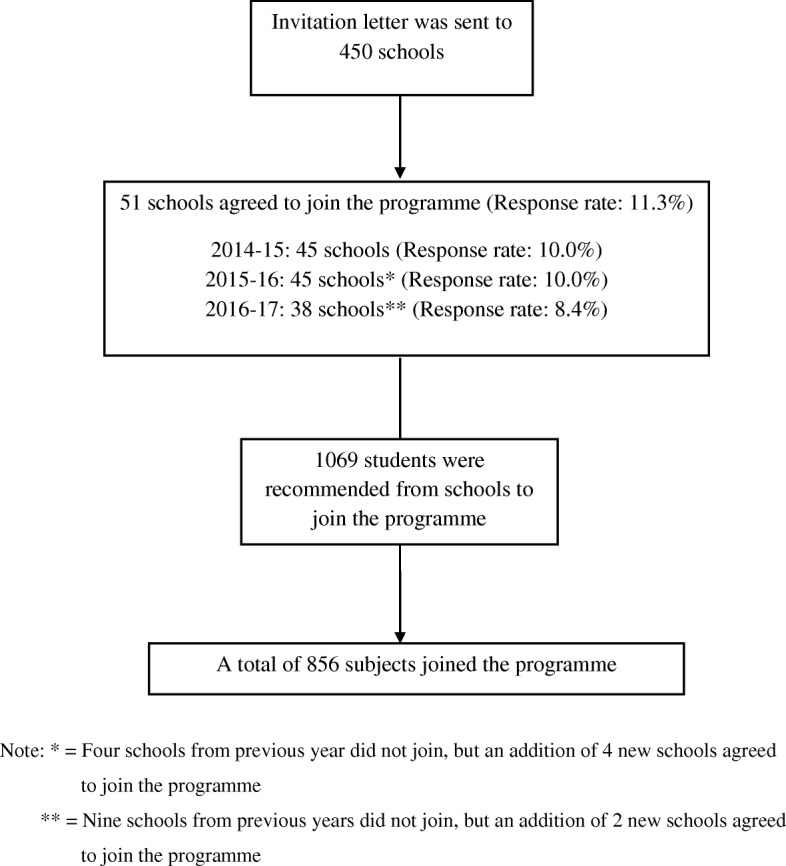
Summary of the recruitment process

All students or teenagers aged 14 to 18 years from secondary schools, youth centres and uniform groups were eligible to join the Smoke-free Teens Programme. The uniform groups referred to any organization with their members wearing uniforms to signify the mission of serving different members in the community [16]. Promotion and recruitment was conducted early in each year and the programme was implemented from July to March of the next year. To recruit eligible participants, invitation letters were sent to around 450 secondary schools every year. For those schools that showed interested to join could contact us via the reply mail. Due to the limited capacity and resources., each school could only nominate around 10 students to join the programme every year. A total of 51 schools agreed to join this programme over the past 3 years. This gave an overall response rate of 11.3%. Figure 1 summarizes the recruitment process. The programme contained three major components. The first component was a 2-day-1-night training camp that aimed to train participants as Smoke-free Teens to promote a smoke-free culture. For the second component, participants were asked to organize creative activities to promote smoke-free messages in their schools and community. The third component included an award presentation ceremony to recognize the efforts of outstanding Smoke-free Teens in establishing a smoke-free culture. To publicize the Smoke-free Teens Programme, COSH produced publicity materials such as posters for display in secondary schools, youth centres, and uniform group meetings. Advertisements were placed in newspapers and magazines, on the radio, television and social media platforms. Programme webpage was set up to promote the programme and allow extended learning for the participants. COSH also organized a series of camp briefing sessions, communication skills training workshops, health talks and seminars. To evaluate the effectiveness of the training programme, a one-group pretest–posttest, within-subjects design was used.

### Details of the programme

#### Smoke-free teens training camp

Approximately four Smoke-free Teens Training Camps were held during each summer holiday. Participants were invited to join the 2-day-1-night training camp between July and August of each year. The camp provided a wide range of adventurous and experiential indoor and outdoor activities, which were implemented by COSH and campsite coaches. In addition, health education talks were delivered by COSH staff and a registered nurse with more than 5 years’ experience in smoking cessation. The talks covered a variety of topics, including the hazardous effects of smoking, tobacco control policies, existing cessation services in Hong Kong, and basic counseling skills, etc. In addition, the participants were taught to deliver brief smoking cessation advice using the AWARD model: ***A***sking about smoking history; ***W***arning about the high risk that one out of every two smokers will be killed prematurely by smoking, which is the mortality risk for smokers in general suggested by World Health Organization; ***A***dvising to quit as soon as possible; ***R***eferring to smoking cessation clinics or hotlines; ***D***oing it again until smokers quit smoking. The AWARD model was developed according to the clinical practice guideline for smoking cessation. It aids quitting by warning smokers of the high mortality risk of smoking and referring those in need of more intensive counseling to smoking cessation services [17]. This model has been tested in our previous studies on smoking cessation. The findings of these studies indicated that this model is effective in helping smokers in community settings quit smoking [17–19]. By engaging in the 2-day-1-night camp activities, participants obtained the latest information about tobacco hazards and tobacco control. To help them effectively design and execute their smoke-free advocacy activities, participants were trained in a broad array of skills, including leadership, creative and critical thinking, communication, problem solving, team building, programme planning, smoking cessation counseling techniques, and social media marketing techniques.

### Promoting smoke-free messages in schools and the community

After completing the training camp, participants were asked to apply the various skills they had learned to design and implement at least one smoke-free activity in their schools or the community during September–December in small groups to promote smoke-free messages. They also encouraged their friends, families, and neighbors to quit smoking and promote the concept of a smoke-free Hong Kong.

### Award presentation ceremony

To commend the outstanding Smoke-free Teens and schools/organizations for their efforts in establishing a smoke-free culture, an award presentation ceremony was held in March of the following year. Smoke-free Teens who had shown outstanding performances were presented with a trophy and book voucher as encouragement. In addition, certificates were presented to Smoke-free Teens who had completed the entire programme.

### Outcomes

To evaluate the Smoke-free Teens Programme, teenagers who participated in the programme between July 2014 and March 2017, and whose parents had signed the consent forms, were included in this evaluation study. The outcome measures were changes in (1) knowledge about smoking hazards; (2) attitudes towards smoking, tobacco control, and smoking cessation; and (3) practices for promoting smoking cessation.

### Data collection

A structured questionnaire was administered by a research assistant at baseline, immediately after the training camp, and 3 and 6 months later to assess changes in participants’ knowledge and attitudes regarding smoking and tobacco control. At baseline, participants were asked whether they had experienced in promoting smoking cessation in the previous 6 months. At 3- and 6-month follow-ups after attending the training camp, participants were asked to report their practice of promoting smoking cessation for the past 3 months.

### Data analyses

We used the Statistical Package for the Social Sciences (SPSS: Version 23; SPSS Inc., Chicago, IL, USA) for Windows to analyze the data. Descriptive statistics were used to analyze participant demographic characteristics. The paired samples t-test and McNemar’s test were used to assess any changes in participants’ knowledge and attitudes regarding smoking and tobacco control before and after the training camp. One-Way Repeated Measures ANOVA was used to assess any changes in participants’ cessation practice before and after the training camp. Descriptive statistics were used to describe the purposes of the smoke-free activities organized by the participants. Missing values were handled by the baseline-observation-carried-forward.

## Results

**Fig. 2:**
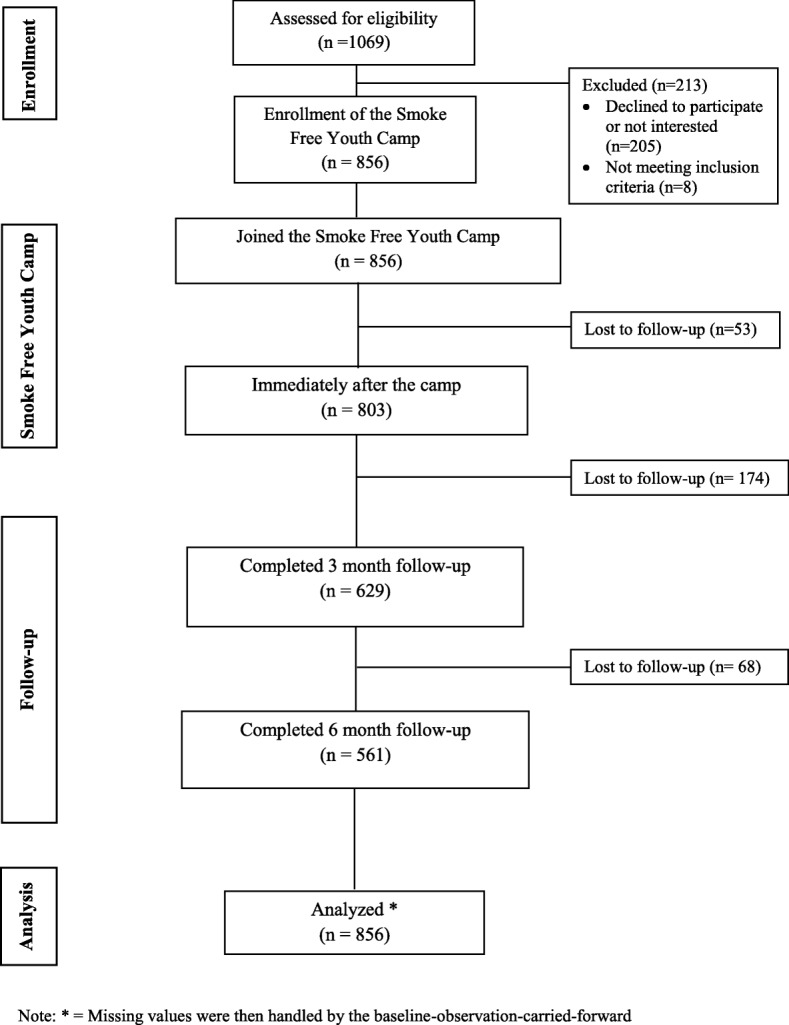
The CONSORT diagram showing how participants flowed in the study

A total of 856 teenagers were recruited during the study period. However, 26.5% (227/856) and 34.5% (295/856) lost to follow-up at 3 and 6 months, respectively. Figure 2 is a CONSORT diagram tracking how the participants flowed in this evaluation study.

Table 1 shows the participant demographic characteristics at baseline. The mean age of the participants was 15.2 (SD = 1.1) years. About 65.0% (556/856) of the participants were female and 33.2% (284/856) were male. About 46.0% (394/856) of the participants were Secondary 4 students and 82.1% (703/856) were never smokers. About 53.5% (458/856) of the participants lived with at least one smoker. Of these participants, 65.9% (302/458) lived with smokers who were their fathers.

**Table 1:** Demographic Characteristics of the Participants (*N* = 856)

Gender, *n* (%)	
Male	284 (33.2)
Female	556 (65.0)
Missing	16 (1.8)
Age, mean (SD^a^)	15.2 (1.1)
Year of study, *n* (%)	
Secondary 1	0 (0.0)
Secondary 2	19 (2.2)
Secondary 3	160 (18.7)
Secondary 4	394 (46.0)
Secondary 5	268 (31.3)
Secondary 6	11 (1.3)
Missing	4 (0.5)
Smoking status, *n* (%)	
Never smoker	703 (82.1)
Current smoker	1 (0.1)
Ex-smoker	12 (1.4)
Missing	140 (16.4)
Living with smoker(s), *n* (%)	
Yes	458 (53.5)
No	393 (45.9)
Missing	5 (0.6)
Living with whom smoker(s), *n* (%^b^)	
Father	302 (65.9)
Mother	59 (12.9)
Brother	18 (3.9)
Sister	10 (2.2)
Grandfather	84 (18.3)
Grandmother	20 (4.4)
Other relatives	165 (36.0)
Others	25 (5.5)

^a^*SD* standard deviation

^b^The percentages were calculated using the total number of the participants who living with smokers (*N* = 458) as dominator

Table 2 shows the changes in knowledge about smoking hazards. When compared with baseline, a significant improvement was observed in knowledge about smoking hazards (58.0% versus 75.0%; *p* = .016; correctly answered all items) at 6-month follow-up. Table 3 shows the changes in attitudes towards smoking cessation and tobacco control. Compared with baseline, significant improvements were observed in several areas at 6 months, including advising their friends to quit smoking (4.43 ± 0.68 versus 4.49 ± 0.61; *p* = .012), asking people not to smoke around them (4.39 ± 0.75 versus 4.50 ± 0.66; *p* < .001), reminding others not to smoke in non-smoking areas (4.12 ± 0.85 versus 4.29 ± 0.77; *p* < .001), supporting expanding non-smoking areas (4.31 ± 1.05 versus 4.50 ± 0.78; *p* < .001), increasing tobacco tax (4.14 ± 0.94 versus 4.39 ± 0.82*; p* < .001), and a total ban of the sale of tobacco products (4.08 ± 0.99 versus 4.43 ± 0.82; *p* < .001). Table 4 shows statistically significant changes in practice towards smoking cessation and tobacco control. Compared with baseline, participants reported that they had provided smoking cessation advice to more smokers (1.6 ± 1.5 versus 2.3 ± 4.3; *p* < .001) at 6-month follow-up. All of them also reported making more smoker referrals to existing smoking cessation services (2.2 ± 2.2 versus 3.3 ± 7.7, *p* < .001) at 6 months.

**Table 2:** Knowledge of Smoking Hazards among the Participants (*N* = 856)

	Baseline	Immediately after the camp		3-month after the camp		6-month after the camp	
	Correct responses *n* (%)	Correct responses *n* (%)	*p*	Correct responses *n* (%)	*p*	Correct responses *n* (%)	*p*
No matter how long a person smokes, quit smoking is never too late (Correct)	687 (80.3)	768 (89.7)	<.001**	759 (88.7)	<.001**	764 (89.3)	<.001**
1 in 2 smokers will be killed by smoking (Correct)	587 (68.6)	829 (96.8)	<.001**	771 (90.1)	<.001**	793 (92.6)	<.001**
Nicotine replacement therapy (NRT) patches & gums can increase the success rate of quitting smoking (Correct)	424 (49.5)	522 (61.0)	<.001**	514 (60.0)	<.001**	516 (60.3)	<.001**
2nd hand smoke is less harmful than outdoor air pollution (Incorrect)	559 (65.3)	547 (63.9)	.577	571 (66.7)	.558	540 (63.1)	.406
After smoking in a house, the residual chemicals left in the air will damage the health of infants and children (Correct)	820 (95.8)	824 (96.3)	.708	817 (95.4)	.710	802 (93.7)	.068
The use of e-cigarettes is allowed in non-smoking areas (Incorrect)	380 (44.4)	579 (67.6)	<.001**	511 (59.7)	<.001**	485 (56.7)	<.001**
	%	%	*p*	%	*p*	%	*p*
Correctly answered all the items	58.0	74.5	.025*	73.7	.025*	75.0	.016*

Missing data or answered “don’t know” were regarded as “incorrect”

* = statistically significant at *p* < 0.05; ** = statistically significant at *p* < 0.001

**Table 3:** Attitudes towards Tobacco Control and Smoking Cessation among the Participants (*N* = 856)

	Baseline	Immediately after the camp		3-month after the camp		6-month after the camp	
	Mean (SD)	Mean (SD)	*p*	Mean (SD)	*p*	Mean (SD)	*p*
I will proactively advise my friends to quit smoking	4.43 (0.68)	4.48 (0.64)	.030*	4.51 (0.60)	<.001**	4.49 (0.61)	.012*
I will ask people not to smoke around me	4.39 (0.75)	4.45 (0.70)	.017*	4.47 (0.67)	.017*	4.50 (0.66)	<.001**
I will remind others that they are not allowed to smoke in non-smoking areas	4.12 (0.85)	4.34 (0.73)	<.001**	4.27 (0.80)	<.001**	4.29 (0.77)	<.001**
I support expanding non-smoking areas	4.31 (1.05)	4.48 (0.79)	<.001**	4.52 (0.78)	<.001**	4.50 (0.78)	<.001**
I support increasing tobacco tax	4.14 (0.94)	4.35 (0.85)	<.001**	4.42 (0.76)	<.001**	4.39 (0.82)	<.001**
I support banning the sale of tobacco products comprehensively	4.08 (0.99)	4.33 (0.89)	<.001**	4.40 (0.83)	<.001**	4.43 (0.82)	<.001**

* = statistically significant at *p* < 0.05; ** = statistically significant at *p* < 0.001

Missing data was excluded

1: Strongly disagree; 5: Strongly agree; *p*-values indicate the use of paired samples t-tests to compare with baseline

**Table 4:** The Results of the One-Way Repeated Measures ANOVA for Practice towards Tobacco Control and Smoking Cessation among the Participants (*N* = 856)

	Mean (SD)			Time effect			
	Baseline	3-month after the camp	6-month after the camp	*F*-value	*p*-value	^a^Eta Squared	Power
Number of smokers given cessation advice by each participant	1.6 (1.5)	1.5 (1.3)	2.3 (4.3)	12.56	<.001*	0.03	1.00
Number of smokers referred to smoking cessation services by each participant	2.2 (2.2)	1.7 (1.2)	3.3 (7.7)	34.82	<.001*	0.08	1.00

^a^Effect size (Eta squared) conventions: small effect = 0.01; moderate effect = 0.06; large effect = 0.14

* = statistically significant at *p* < 0.001

Over the course of the 3-year study, Smoke-free Teens organized 552 smoke-free programmes, with each group conducting 3.2 programmes on average. These programmes reached 144,528 people in schools and the community. The highly diverse programmes included among the many activities exhibitions, mosaic art and microfilm production, game booths, debate and poster design competitions, as well as the promotion of a smoke-free culture on busy streets. An outstanding example was the team that won the 2014 championship. It organized a wide range of activities including a slogan competition, game booths, an anti-cancer health talk, and video production. To enforce the smoke-free message to primary school students and the public, they also invited people to make a smoke-free pledge, distributed bookmarks and cards with smoking cessation hotline numbers to smokers, as well as hung a banner with a smoke-free slogan at a minibus terminal. Over 2000 citizens were reached by their activities.

Table 5 shows the aim of the smoke-free activities organized by the participants. Of the organized smoke-free activities, 73.6% (406/552) publicized smoke-free messages, 61.4% (339/552) educated the public about the hazardous effects of smoking and secondhand smoke exposure, 35.3% (195/552) promoted smoking cessation, 1.6% (9/552) introduced our training programme to the public, and 0.7% (4/552) discussed the economic losses caused by smoking.

**Table 5:** Aim of the Smoke-free Activities Organized by the Participants

Aim	Total = 552
	*n* (%)^a^
Promotion of “smoke-free” messages	406 (73.6)
Education on the hazardous effects of smoking and secondhand smoke exposure	339 (61.4)
Promotion of smoking cessation	195 (35.3)
Introduction of the Smoke-free Teens Programme	9 (1.6)
Discussion on the economic losses caused by smoking	4 (0.7)

^a^The percentages were calculated using the total number of the smoke-free activities as dominator

## Discussion

Youth smoking is a global public health concern that has been regarded as a “pediatric epidemic” [20]. Building an awareness of tobacco control and smoking cessation among youths is very important in reducing smoking initiation, which in turn can lower the prevalence of cigarette use. This paper reports a study to promote a smoke-free lifestyle and smoking cessation in the community by mobilizing teenagers from secondary schools, youth centres, and uniform groups to serve as smoke-free ambassadors. In addition, we evaluated the effectiveness of the training programme by assessing changes in knowledge, attitudes, and practices of tobacco control and cessation among the ambassadors.

The present study demonstrated that the training programme was effective in enhancing the knowledge of smoking hazards among youths. This is evidenced by the fact that 75.0% of participants were able to correctly answer all items at 6-month follow-up compared with only 58.0% at baseline. Moreover, there were statistically significant improvements in participants’ attitudes towards tobacco control policies, such as expanding non-smoking areas, increasing tobacco tax, and a total ban of the sale of tobacco products. In addition, the results supported the effectiveness of the training programme in changing Smoke-free Teens’ practice of smoking cessation promotion. There were statistically significant increase in the mean number of smokers who received smoking cessation advice and who were referred to smoking cessation services by participants at 6-month follow-up compared with baseline. However, caution must be taken when interpreting these findings. The results might be highly confounded by the number of smokers that the participants had encountered before and during the study. Furthermore, the results could be misleading if the participants only encountered a few smokers at baseline, but more smokers during the period of evaluation. Nevertheless, the results demonstrated that the trained Smoke-free Teens could successfully promote cessation among smokers using the AWARD model. One advantage of the AWARD model is that it can be easily learned and used by teenagers with minimal training. In addition, it takes only a minute or slightly longer to communicate advice based on the AWARD model. This makes it very useful in promoting smoking cessation in community settings and permits a sizeable number of smokers to be reached at low cost.

Following the intensive training, the Smoke-free Teens formed into groups and applied the various skills they had learned to design and implement creative projects to disseminate smoke-free messages in their schools or the community. During the study period, 552 smoke-free activities were held, which reached 144,528 people. These smoke-free activities were effective in raising public awareness about smoking hazards and obtaining support for public health policies. In addition, the activities allowed the promotion of smoke-free messages to a segment of smokers who are difficult to be reached by existing smoking cessation services. When compared with other methods in health promotion, such as educational talks and self-help materials, these activities are a practical and effective way to rapidly disseminate health messages to a large number of people.

We received much positive feedback from the trained Smoke-free Teens. One group of Smoke-free Teens commented that they found the experience of organizing such a large-scale event valuable. They felt that the experience helped them realize the importance of group unity and planning, and that the activities had fully utilized the potential of every teammate. They hoped that everyone might develop a smoke-free healthy lifestyle. Most importantly, by joining this programme, participants were not only equipped with knowledge about tobacco control and smoking hazards, but were also more likely to refrain from trying smoking in the future. One trained Smoke-free Teen of the 2016–2017 champion team stated that “I spent half a year planning and organizing the smoke-free activities and promoting a smoke-free lifestyle. I never regretted joining the programme, as what I learned and experienced was invaluable and could not be acquired at school. It is a very precious memory. Besides, my father reduced his tobacco consumption with my support and encouragement.”

There are several benefits in mobilizing secondary school students to serve as ambassadors of smoking cessation in the community. First, they may encounter smokers in their social circles. Young smokers, particularly those who are reluctant to access smoking cessation services, may be more willing (and may find it easier) to receive brief advice on smoking cessation from the ambassadors owing to the established rapport between them. Second, there are more than 300,000 secondary school students in Hong Kong [21]. Trained Smoke-free Teens could play an important role in helping to widely disseminate smoke-free messages in the community. In particular, they can motivate their smoking peers to quit by being healthy role models.

Despite only small changes in participants’ knowledge and attitudes regarding smoking and tobacco control after the programme, it is meaningful and relevant to public health practice. In particular, within a 6-month period of evaluation, 856 ambassadors were able to deliver brief cessation advice to 1968 smokers in their social circles. The ambassadors also referred 2824 smokers to smoking cessation services. It was expected that the ambassadors would continue such practice after the evaluation. The running cost of this programme is low in comparison to the healthcare cost resulted from treating tobacco-related diseases of a smoker. Therefore, this innovative, inexpensive and sustainable approach can make an important contribution to establishing a smoke-free culture in schools and the community in Hong Kong, consequently saving more lives.

### Limitations

There were some limitations of this study. Frist, this study was limited because only 51 out of 450 schools joined the Smoke-free Teens Programme over the past 3 years. The low response rate might be due the Hong Kong education system is exam-oriented and the schools in Hong Kong are highly academic-oriented. Majority schools in Hong Kong are mainly focused on academic results, but neglect the importance of having extra-curricular activities and building students’ personal characters. Consequently, students are busy with attending different tutorial classes after school but no more extra time for joining activities. Given the significant health impact of the Smoke-free Teens Programme on our new generation, more resources and efforts should be allocated to this area to create a rapport with schools and parents with regard to tobacco control advocacy. The second limitation was that we did not follow up smokers who had received brief smoking cessation advice from Smoke-free Teens, and hence could not evaluate the long-term impact of the training programme in the community, particularly in terms of smoking cessation outcomes. Studies with longer follow-ups are therefore warranted to examine the sustainability of the training effects. Furthermore, a comprehensive evaluation is needed to determine whether the training effects are translated into smoking cessation at an individual level. Additional measures, including (1) how many family members and friends are invited to make a quit attempt, (2) whether local quitlines receive more referrals, and (3) whether friends of ambassadors are more willing to receive cessation advices from the ambassadors in comparison to other sources could be assessed in future evaluation. The third limitation was that participation in this study was on a voluntary basis. It was expected that participants and their families were more negative towards cigarette smoking, consequently might bias the results. Despite the family background might influence the participants’ attitudes towards smoking and their smoking status [22], we did not observe any significant difference in these two variables with different family backgrounds in subgroup analyses. Future studies with more stringent design are necessary to confirm our findings.

#### Implications for practice

The findings from this project have important implications for future practice. The project enhanced the community’s capacity to promote smoking cessation because there was a significant improvement in the knowledge, attitudes, and smoking cessation practices of the trained Smoke-free Teens. Moreover, trained Smoke-free Teens can continue to help promote smoking cessation after training. Participants were encouraged to join the Smoke-free Teens Alumni Programme and they continue to promote a smoke-free message by attending sharing sessions, managing game booths and exhibitions in the community, and participating in other tobacco control activities organized by COSH. The World Health Organization [23] emphasizes the importance of member states implementing multisectoral control measures against the tobacco industry. By joining the Smoke-free Teens Programme, the activities of trained Smoke-free Teens could become one of the main ways of implementing the government’s tobacco control policies in Hong Kong.

## Conclusions

Notwithstanding some limitations, the overall results of this study suggest the effectiveness of the training programme in changing knowledge about smoking hazards, attitudes towards smoking, and tobacco control and smoking cessation practices. It is encouraging that the trained Smoke-free Teens not only promoted smoke-free messages among their schoolmates, friends, and families, but also began to gather community support for a smoke-free Hong Kong. The Smoke-free Teens Programme has been instrumental in fostering a new batch of Smoke-free Teens to advocate a smoke-free culture and protect the health of the public.

## Abbreviations

AWARD: Ask, Warn, Advise, Refer and Do-it-again
COSH: The Hong Kong Council on Smoking and Health
